# Pharmacovigilance Signal Detection and Mutually Exclusive Driver Mutations of the PI3K/AKT Pathway in Breast Cancer Treated With Capivasertib

**DOI:** 10.1155/humu/9532614

**Published:** 2026-06-25

**Authors:** Zhanyang Luo, Yi Shi, Bukun Zhu, Qionglian Huang, Wei Zhang, Youyang Shi, Xingchen Yang

**Affiliations:** ^1^ Department of Pharmacy, Longhua Hospital, Shanghai University of Traditional Chinese Medicine, Shanghai, China, shutcm.edu.cn; ^2^ Department of Pharmacy, Shanghai Pudong Hospital, Fudan University Pudong Medical Center, Shanghai, China, fudan.edu.cn; ^3^ Suzhou Hospital, Affiliated Hospital of Medical School, Nanjing University, Suzhou, China, nju.edu.cn; ^4^ Department of Infection, Longhua Hospital, Shanghai University of Traditional Chinese Medicine, Shanghai, China, shutcm.edu.cn; ^5^ Institute of Chinese Traditional Surgery, Longhua Hospital, Shanghai University of Traditional Chinese Medicine, Shanghai, China, shutcm.edu.cn

**Keywords:** breast cancer, capivasertib, molecular dynamics, pharmacovigilance, single-cell RNA sequencing, tumor microenvironment

## Abstract

**Background and Objective:**

This study is aimed at comprehensively evaluating the real‐world safety profile and underlying molecular mechanisms of the AKT inhibitor capivasertib by integrating pharmacovigilance data, computational biology, and multiomics analyses.

**Methods:**

Adverse event (AE) reports from the FAERS database were analyzed using disproportionality algorithms (ROR, PRR, BCPNN, EBGM) to detect significant safety signals. To elucidate potential toxicological mechanisms, we employed network toxicology and molecular docking, further validated by 100 ns molecular dynamics (MD) simulations. Additionally, bulk genomic cohorts (e.g., TCGA, METABRIC) and single‐cell RNA sequencing (scRNA‐seq) datasets were utilized to assess mutation patterns and delineate key targets within the tumor microenvironment (TME).

**Results:**

Analysis of 22,143 AE reports yielded 133 significant safety signals, predominantly involving metabolism (e.g., hyperglycemia), the gastrointestinal system (e.g., nausea, stomatitis), and dermatological conditions (e.g., rash). Pathway enrichment highlighted the PI3K‐AKT, HIF‐1, and EGFR signaling networks. Integrative analyses identified critical toxicity‐related modulators, notably AKT1, IGF1, PTEN, TP53, and GSK3B. Crucially, MD simulations robustly confirmed the thermodynamic stability of the capivasertib–GSK3B complex. Genomic profiling revealed pronounced mutual exclusivity among PIK3CA, AKT1, and PTEN alterations. Furthermore, scRNA‐seq analysis demonstrated that GSK3B overexpression defines a highly aggressive, proliferative malignant subpopulation that profoundly reshapes intercellular communication with stromal fibroblasts.

**Conclusion:**

By seamlessly bridging real‐world pharmacovigilance with advanced structural biology and single‐cell transcriptomics, this study delineates the comprehensive safety landscape of capivasertib. Our findings provide crucial clinical alerts for AE monitoring and offer deep mechanistic insights to optimize personalized therapeutic management in breast cancer.

## 1. Introduction

Breast cancer (BC) remains the most frequently diagnosed malignancy and a leading cause of cancer‐related mortality among women worldwide. The hormone receptor–positive, HER2‐negative (HR+/HER2−) subtype constitutes the most common form, for which endocrine therapy is the mainstay of treatment. However, the development of resistance to ET—particularly during Cyclin‐dependent kinases 4/6 inhibitor administration—represents a persistent therapeutic challenge [1]. Capivasertib, an oral, selective ATP‐competitive inhibitor of AKT1/2/3, has shown promise in tumors with aberrations in the PI3K/AKT/PTEN signaling pathway [2].

In November 2023, the FDA approved capivasertib as a treatment option for adults who have HR+, advanced or metastatic HER2− BC with a mutation in the PIK3CA gene, AKT1 gene, or PTEN gene that has progressed while on (or within 6 months of discontinuation of) treatment with at least one line of systemic endocrine therapy [3].

Although clinical activity of capivasertib is promising, there are no data regarding real‐life safety; the AEs with highest frequency seen in trials include diarrhea, skin responses, hyperglycemia and elevated liver enzymes [4], but such RCTs may not be representative of rare, late‐onset, or age‐related effects. Therefore, postmarket pharmacovigilance is necessary to fully understand the risk profile of a drug and it has been widely recognized that spontaneous reporting systems are one of key sources of real‐world information on adverse events: FDA Adverse Event Reporting System (FAERS) from the US Food and Drug Administration (FDA) allowing the identification of risks and problems that might not be identified during the preapproval review process [5]. Furthermore, there are new approaches in the area of network toxicology that combines computer biology and chemical information science and target discovery for exploring potential molecular mechanisms of drug toxicity [6, 7].

In the present work, we combined pharmacovigilance and network toxicology methods aiming at studying the adverse events profile related to the drug capivasertib. According to data available into FAERS, we conducted disproportionality analyses to detect possible safety signal events and then used the results for further analysis based on network pharmacology theory to explore putative cellular and molecular targets of drug toxicity. This integrative strategy provides real‐world safety evidence and mechanistic insights to support the rational clinical use of capivasertib in BC treatment.

## 2. Materials and Methods

### 2.1. Data Source and Extraction

AE reports for capivasertib were extracted from the FAERS (https://fis.fda.gov/extensions/FPD-QDE-FAERS/FPDQDE-FAERS.html), covering the period from Q1 2004 to Q1 2025. Data was obtained via OpenVigil 2.1 (https://h2876314.stratoserver.net:8080/OV2/search), an open‐source pharmacovigilance platform. Reports listing capivasertib as a suspect (primary or secondary) or interacting drug were included. Duplicate entries were identified and removed based on FDA deduplication guidance to ensure unique case counts. Drug names were standardized to generic forms, and AEs were mapped to Preferred Terms (PTs) according to the Medical Dictionary for Regulatory Activities (MedDRA) Version 24.1. Data preprocessing, including filtering and aggregation, was conducted using R (R Foundation for Statistical Computing, Vienna, Austria) and SQL‐based scripts.

### 2.2. Network Toxicology Analysis

To investigate the potential toxicological mechanisms of capivasertib, we employed a network toxicology approach integrating multiple public databases. Putative drug targets were retrieved from SwissTargetPrediction (http://www.swisstargetprediction.ch/), DGIdb database (https://www.dgidb.org/), DisGeNET database (https://www.disgenet.com/), CTD database (https://ctdbase.org/), ChEMBL database (https://www.ebi.ac.uk/chembl/), and STITCH database (http://stitch.embl.de/) [8]. Toxicity‐ and disease‐related genes were obtained from GeneCards. Intersection analysis was performed to identify overlapping genes, representing potential toxicity‐related targets of capivasertib.

These intersecting genes were input into the STRING database to construct a protein–protein interaction (PPI) network, which was subsequently visualized and analyzed using Cytoscape. The essential genes were identified based on the analysis of topological features. To further explain key biological function and signal pathways possibly related with toxicity caused by capivasertib, we carried out functional enrichment analysis, including Gene Ontology (GO) term and Kyoto Encyclopedia of Genes and Genomes (KEGG) analysis based on the DAVID database online tool.

### 2.3. Molecular Docking

The SMILES format of drug molecule capivasertib is obtained by downloading it from PubChem database (https://pubchem.ncbi.nlm.nih.gov/) [9], whereas the 3D structure of the main target protein was obtained from the RCSB Protein Data Bank (https://www.rcsb.org/) [10]. The initial preparation of proteins included removal of water molecules, as well as adding hydrogen atoms using PyMOL and AutoDock Tools programs. The drug molecule as well as protein was converted into PDBQT file for performing docking simulation. Docking simulation was performed by using AutoDock Vina, docking studies were carried out to predict best fit pose, as well as affinity score for the interaction between capivasertib with receptor protein. Docking accuracy was validated by standard protocol controls, and the binding energy of each complex was calculated to evaluate interaction strength and stability.

### 2.4. Molecular Dynamics (MD) Simulations

MD simulations of the capivasertib‐GSK3B complex were conducted using GROMACS Version 2022.2. The protein topology was generated using the Amber14sb force field, whereas the ligand was parameterized with the general Amber force field (GAFF) incorporating restrained electrostatic potential (RESP) charges. The complex was centered in an octahedral simulation box, solvated with the TIP3P water model, and neutralized by adding an appropriate number of Na + /Cl − ions.

The system was initially subjected to energy minimization using the steepest descent algorithm to eliminate steric clashes. Subsequently, equilibration was performed in the NVT (constant number of particles, volume, and temperature) and NPT (constant number of particles, pressure, and temperature) ensembles with position restraints. The system temperature was coupled to 300 K, and pressure was maintained at 1 bar. Long‐range electrostatic interactions were computed using the particle mesh Ewald (PME) method, and all covalent bonds were constrained utilizing the LINCS algorithm.

Following equilibration, a 100‐ns production MD simulation was executed without position restraints. The structural stability and dynamic behavior of the complex were evaluated by extracting trajectories to calculate the root‐mean‐square deviation (RMSD), root‐mean‐square fluctuation (RMSF), radius of gyration (RoG), solvent‐accessible surface area (SASA), and the number of intermolecular hydrogen bonds. Furthermore, to identify the thermodynamically most favorable binding state, 2D and 3D Gibbs free energy landscapes (FELs) were constructed by projecting the trajectory data onto the RMSD and RoG reaction coordinates.

### 2.5. Single‐cell RNA Sequencing Analysis

The scRNA‐seq dataset comprising 94,119 cells from 26 BC samples was retrieved from the Gene Expression Omnibus (GEO) under accession number GSE176078 [11]. Data preprocessing and downstream analyses were performed using the Seurat R package (V4.0). Quality control filtering retained cells with 200–7500 detected genes, > 500 unique molecular identifiers (UMIs), and < 15% mitochondrial gene content. After log‐normalization and selection of the top 2000 highly variable genes, PCA was performed and the first 30 principal components were used for uniform manifold approximation and projection (UMAP) and graph‐based clustering (resolution = 0.8), yielding nine major cell types annotated by established lineage‐specific markers. GSK3B expression was projected onto the UMAP embedding to assess its spatial distribution within epithelial and malignant compartments. Malignant epithelial cells were stratified into GSK3B‐positive (GSK3B^+^ Malig) and GSK3B‐negative (GSK3B^−^ Malig) subsets based on GSK3B transcript detection. Differential expression analysis was performed using the Wilcoxon rank‐sum test, followed by Gene Set Enrichment Analysis (GSEA) of MSigDB Hallmark pathways [12]. Pseudotime trajectory inference was conducted with Monocle 2 [13]; ordering genes were identified by differential GeneTest (*q* < 0.01), and dimensionality reduction was performed using the DDRTree algorithm to partition malignant cells into five discrete states. High‐dimensional weighted gene coexpression network analysis (hdWGCNA) was applied to malignant cells to identify transcriptional modules (M1–M6) and their hub genes [14]. Cell–cell communication networks were inferred using CellChat [15] with the human CellChatDB ligand‐receptor database, and communication probabilities and interaction strengths were compared between GSK3B^+^ and GSK3B^−^ malignant cells. Statistical significance was assessed using the Wilcoxon rank‐sum test or chi‐squared test as appropriate.

### 2.6. Data Acquisition and Mutation Analysis

To investigate the molecular landscape of the PI3K/AKT/PTEN pathway, somatic mutation data from BC patients were retrieved from cBioPortal for Cancer Genomics (http://www.cbioportal.org) (22588877). Four independent cohorts were analyzed: the METABRIC study (22522925, 30867590, 27161491), the MSK BC cohort (40379787), the TCGA Breast Invasive Carcinoma dataset (26451490), and The Metastatic Breast Cancer Project. Mutation data were downloaded and processed to identify genetic alterations in PIK3CA, AKT1, AKT2, AKT3, and PTEN. The oncoprint plot was generated to visualize the distribution and types of genetic alterations across the five genes of interest. Mutations were categorized by functional consequences and driver status, including missense mutations, truncating mutations, inframe mutations, splice mutations, and structural variants, with further classification as putative driver or unknown significance. Copy number alterations were annotated as amplification (red) or deep deletion (blue). Samples with no alterations or not profiled were indicated in gray and white, respectively. The percentage of altered cases for each gene was calculated based on the total number of profiled patients. Lollipop plots were constructed to illustrate the distribution of mutations along the protein domains of PIK3CA, AKT1, and PTEN. Protein domain annotations were retrieved from UniProt and Pfam databases. The vertical position of each lollipop represents the number of patients harboring mutations at specific amino acid residues, with color coding indicating mutation types. Hotspot mutations were highlighted with detailed amino acid change annotations. The *x*‐axis denotes amino acid positions, and colored blocks represent functional protein domains: PI3K_p85B, PI3K_rbd, PI3K_C2, PI3Ka, and PI3_PI4_kinase domains for PIK3CA; PH, Pkinase, and Pkinase_C domains for AKT1; and DSPc and PTEN_C2 domains for PTEN. Statistical analysis of mutual exclusivity and co‐occurrence between gene pairs was performed using Fisher′s exact test. For each gene pair (A and B), patients were classified into four categories: neither gene mutated, only Gene A mutated (A not B), only Gene B mutated (B not A), and both genes mutated (both). log_2_ odds ratios were calculated to quantify the direction and magnitude of association. *p* values were computed using two‐sided Fisher′s exact test and adjusted for multiple comparisons using the Benjamini–Hochberg method to generate *q* values. Gene pairs with *q* < 0.05 were considered statistically significant. Mutual exclusivity was defined as log_2_ odds ratio < 0 with significant *q* value, whereas co‐occurrence was defined as log_2_ odds ratio > 0 with significant *q* value.

### 2.7. Signal Detection and Statistical Analysis

For each AE of interest and for all drug‐related AEs associated with capivasertib, investigators used four well‐established disproportionality measures including reporting odds ratio (ROR), proportional reporting ratio (PRR), Bayesian confidence propagation neural network (BCPNN), and empirical Bayes geometric mean (EBGM) [16]. These identify whether any AEs occurred more frequently in patients treated with capivasertib than they did overall across all other drug products in the database. Each of the drugs was combined with all events to construct a 2 × 2 table; then ROR and PRR, together with their 95% CIs were calculated using standard methods. Detailed results from the statistics analysis are presented in Table S1 and Table S2. To calculate ICs for use in our BCPNN model and EBGM values we used MGPS: using the OpenVigil software package.

Ae was defined to be important if it met any of the following predefined criteria: ROR ≥ 1, 95*%*CI lower limit > 1, and ≥ 3 reports; a RRR ≥ 2 with corresponding *χ* ^2^ ≥ 4; Information Component 025 (IC 025) > 0 (lower 95% credibility interval); or EB05 > 2 (lower 95% credibility interval of EBGM) [16]. All statistics are computed in R and we only report on those signals that have been detected by more than one method for higher specificity). We report all detected signals at PT‐level, with appropriate statistics (and 95% CI) for further analysis and discussion.

## 3. Results

### 3.1. Descriptive Analysis of Capivasertib AE Reports

**Figure 1:**
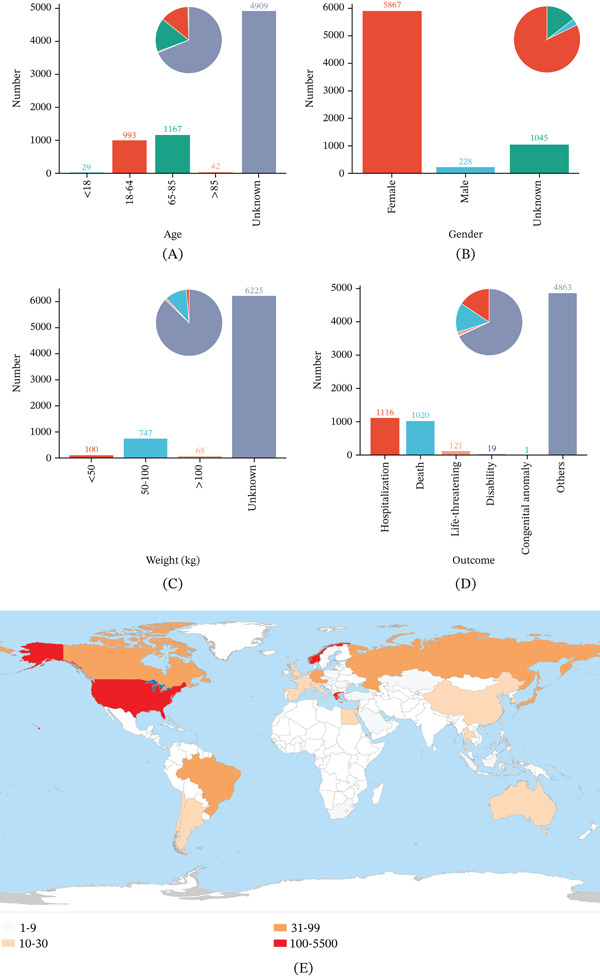
Demographic characteristics of AE reports for capivasertib. (A) distribution of age, (B) gender, (C) body weight, (D) reported outcome, and (E) country/region of capivasertib AE reports.

To explore AEs associated with capivasertib, we queried AE reports in FAERS between November 2023 and March 2025. In total there were 22,143 reported cases (female: 82.17%), as this drug is mainly used for BC, whereas only 3.19% were men (Figure 1). After excluding 4909 observations due to missing data on patients′ ages, it was observed that AEs occurred more often in people aged from 18 up to 64 years old (13.91%) as well as for people aged from 65 to 85 years old (16.34%).

We had body mass for some subset of patients, however ranging from below 50 to above 100 kg; most of the patients have weights in range of 50–100 kg (*n* = 747), next were less than 50 kg (*n* = 100); and greater than 100 kg (*n* = 68). Outcome variable here we can see that major outcome like admission to hospital (*n* = 15.63*%*); deaths (14.29%), severe injuries (1.69%) and lasting disabilities (0.27%). Notably, 68.11% of reports lacked sufficient outcome documentation.

Geographic information showed that most cases were reported in the United States (88.05%), followed by Russia (1.56%) and Canada (1.46%). But, with many reports missing demographic information and outcome status, therefore these results should be interpreted with caution. Summary statistics are provided in Table 1.

**Table 1:** Baseline information on AEs related to capivasertib in the FAERS database.

Variables	Value
Gender	
Female	5867 (82.17%)
Male	228 (3.19%)

Age (years)	
< 18	29 (0.41%)
18–64	993 (13.91%)
65–85	1167 (16.34%)
> 85	42 (0.59%)
Unknown	4909 (68.75%)

Weight (kg)	
< 50	100 (1.40%)
50–100	747 (10.46%)
> 100	68 (0.95%)
Unknown	6225 (87.18%)

Outcome	
Hospitalization	1116 (15.63%)
Death	1020 (14.29%)
Life‐threatening	121 (1.69%)
Disability	19 (0.27%)
Congenital Anomaly	1 (0.01%)
Others	4863 (68.11%)

Note: Categorical variables are presented as *n* (%).

### 3.2. System Organ Class Distribution of Capivasertib AEs

**Figure 2:**
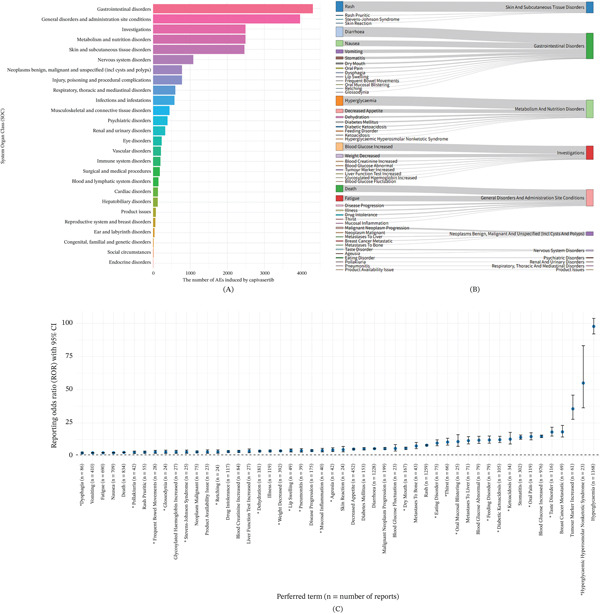
Distribution of positive AE signals at SOC and PT level. (A) The number of PT and AE reports contained in the SOC associated with positive AE signal of capivasertib. (B) Sankey diagram of the relationship between positive AE signal and SOC. (C) Positive AE signals of capivasertib at PT level, including the report number and ROR values. Error bars represent the 95% confidence interval of the ROR. ∗ indicates AE signals not mentioned by the package insert.

A total of 26 system organ classes (SOCs) categories were associated with a AE in the capivasertib pharmacovigilance study, with GI disorders being the most frequent (*n* = 4305, 19.44%), adverse events and other complications of medical care (*n* = 3960, 17.88%), abnormal laboratory values (*n* = 2490, 11.25%), nutrition disorders (*n* = 2489,11.24%), and skin‐related adverse events (*n* = 2462, 11.12%) (Figure 2A,B).

The strongest association was observed among the following two groups of AEs: Gastrointestinal disorders (ROR = 2.60; PRR = 2.29; IC = 1.19; Metabolic/nutritional anomalies (ROR = 5.74, PRR = 5.21, IC = 2.38, EBGM = 5.20), and Congenital anomalies of the musculoskeletal system (ROR = 2.29, PRR = 2.21, IC = 2.38, EBGM = 2.29). All four measures indicated these two domains as statistically significant at a similar level (Table 2).This is consistent with adverse events reported for this drug′s labeling and confirms that the FAERS‐based SDR algorithm can identify a true positive.

**Table 2:** System organ class detection for capivasertib associated adverse events.

System organ class (SOC)	The report number	ROR (95% CI)	PRR (*χ* ^2^)	EBGM (EBGM05)	IC (IC025)
Metabolism and nutrition disorders∗	2489	5.74 (5.51–5.99)	5.21 (8632.82)	5.2 (5.02)	2.38 (2.32)
Gastrointestinal disorders∗	4305	2.6 (2.51–2.69)	2.29 (3411.1)	2.29 (2.22)	1.19 (1.15)
Blood and lymphatic system disorders	147	0.39 (0.33–0.46)	0.39 (141.63)	0.39 (0.34)	−1.35 (−1.59)
Respiratory, thoracic and mediastinal disorders	600	0.56 (0.52–0.61)	0.58 (196.86)	0.58 (0.54)	−0.8 (‐0.92)
General disorders and administration site conditions	3960	1.03 (1–1.07)	1.03 (3.18)	1.03 (1)	0.04 (−0.01)
Infections and infestations	577	0.48 (0.44–0.52)	0.49 (315.48)	0.49 (0.46)	−1.02 (−1.14)
Nervous system disorders	1085	0.56 (0.53–0.59)	0.58 (358.78)	0.58 (0.55)	−0.78 (−0.87)
Investigations	2490	1.94 (1.86–2.02)	1.84 (1008.02)	1.83 (1.77)	0.88 (0.81)
Neoplasms benign, malignant and unspecified (incl cysts and polyps)	782	1.37 (1.28–1.47)	1.36 (76.12)	1.36 (1.28)	0.44 (0.34)
Injury, poisoning and procedural complications	782	0.31 (0.29–0.34)	0.34 (1137.5)	0.34 (0.32)	−1.57 (−1.67)
Skin and subcutaneous tissue disorders	2462	2.19 (2.1–2.28)	2.06 (1410)	2.05 (1.98)	1.04 (0.98)
Musculoskeletal and connective tissue disorders	445	0.37 (0.34–0.41)	0.39 (458.46)	0.39 (0.36)	−1.37 (−1.51)
Renal and urinary disorders	328	0.78 (0.7–0.87)	0.78 (20.05)	0.78 (0.71)	−0.35 (−0.51)
Vascular disorders	213	0.45 (0.39–0.51)	0.45 (143.99)	0.45 (0.4)	−1.14 (−1.34)
Reproductive system and breast disorders	62	0.32 (0.25–0.41)	0.32 (91.2)	0.32 (0.26)	−1.65 (−2.01)
Cardiac disorders	132	0.22 (0.19–0.27)	0.23 (352.29)	0.23 (0.2)	−2.13 (−2.38)
Psychiatric disorders	391	0.3 (0.27–0.34)	0.32 (613.32)	0.32 (0.29)	−1.66 (−1.81)
Social circumstances	14	0.13 (0.08–0.23)	0.13 (78.02)	0.13 (0.09)	−2.89 (−3.63)
Eye disorders	234	0.53 (0.46–0.6)	0.53 (98.71)	0.53 (0.48)	−0.91 (−1.1)
Immune system disorders	200	0.81 (0.71–0.93)	0.81 (8.52)	0.81 (0.73)	−0.3 (−0.5)
Ear and labyrinth disorders	38	0.4 (0.29–0.54)	0.4 (35)	0.4 (0.3)	−1.33 (−1.8)
Hepatobiliary disorders	127	0.62 (0.52–0.74)	0.62 (29.04)	0.62 (0.54)	−0.68 (−0.94)
Surgical and medical procedures	182	0.6 (0.52–0.7)	0.61 (47.1)	0.61 (0.54)	−0.72 (−0.94)
Product issues	75	0.2 (0.16–0.26)	0.21 (230.74)	0.21 (0.17)	−2.27 (−2.6)
Endocrine disorders	7	0.12 (0.06–0.26)	0.12 (43.63)	0.12 (0.07)	−3.02 (−4.04)
Congenital, familial and genetic disorders	16	0.23 (0.14–0.38)	0.23 (40.99)	0.23 (0.15)	−2.11 (−2.81)

*Note:* ∗ indicates positive signals across all four disproportional algorithms.

Abbreviations: 95% CI, two‐sided for ROR; CI, confidence interval; EBGM, the empirical Bayes geometric mean; EBGM05 and IC025 lower one‐sided for EBGM and IC; PRR, proportional reporting ratio; ROR, reporting odds ratio; and *χ* ^2^, chi‐squared.

### 3.3. PT‐Level AE Frequency and Disproportionality Analysis

Following deduping of documents, there were 22,143 documents in total for capivasertib. Of these, by Preferred Term: the use of four different disproportionality metrics (ROR, PRR, BCPNN, and EBGM), identified 133 significant safety signals (shown in Table 3). The five most frequently reported AEs were hyperglycemia (*n* = 2144, 9.68%), rash (*n* = 1259, 5.69%), diarrhea (*n* = 1228, 5.55%), nausea (*n* = 709, 3.20%), and fatigue (*n* = 690, 3.12%) (Figure 2C).

**Table 3:** The number of AE reports and positive signal detection results of capivasertib extracted from FAERS.

PT	The report number	ROR (95% CI)	PRR (*χ* ^2^)	EBGM (EBGM05)	IC (IC025)
Rash	1259	8.19 (7.74–8.67)	7.79 (7477.78)	7.76 (7.4)	2.96 (2.87)
Diarrhea	1228	5.66 (5.35–6)	5.41 (4446.34)	5.4 (5.14)	2.43 (2.35)
Hyperglycemia	1168	97.63 (91.95–103.66)	92.53 (102141.89)	89.35 (84.98)	6.48 (6.39)
Blood glucose increased	976	14.87 (13.94–15.85)	14.25 (11999.67)	14.18 (13.44)	3.83 (3.73)
Death	834	2.81 (2.62–3.01)	2.74 (936.54)	2.74 (2.59)	1.46 (1.35)
Nausea	709	2.58 (2.4–2.78)	2.53 (664.38)	2.53 (2.38)	1.34 (1.23)
Fatigue	690	2.55 (2.36–2.75)	2.5 (626.72)	2.5 (2.34)	1.32 (1.21)
Decreased appetite	452	5.31 (4.84–5.83)	5.22 (1544.84)	5.21 (4.82)	2.38 (2.24)
Vomiting	410	2.5 (2.27–2.76)	2.47 (361.7)	2.47 (2.28)	1.3 (1.16)
∗Weight decreased	392	3.98 (3.6–4.4)	3.93 (857.97)	3.92 (3.61)	1.97 (1.83)
Stomatitis	302	14.19 (12.66–15.9)	14.01 (3631.56)	13.94 (12.67)	3.8 (3.63)
Malignant neoplasm progression	199	5.71 (4.97–6.57)	5.67 (764.57)	5.66 (5.03)	2.5 (2.3)
∗Dehydration	181	3.79 (3.28–4.39)	3.77 (368.66)	3.77 (3.33)	1.91 (1.7)
Disease progression	175	4.21 (3.63–4.89)	4.18 (424.27)	4.18 (3.69)	2.06 (1.84)
∗Dry mouth	167	5.88 (5.05–6.85)	5.84 (669.62)	5.83 (5.13)	2.54 (2.32)
Diabetes mellitus	153	5.53 (4.72–6.49)	5.5 (562.74)	5.49 (4.81)	2.46 (2.22)
∗Oral pain	119	14.73 (12.29–17.64)	14.65 (1505.96)	14.58 (12.53)	3.87 (3.6)
Illness	119	3.88 (3.24–4.65)	3.87 (253.14)	3.86 (3.32)	1.95 (1.69)
Drug intolerance	117	3.47 (2.89–4.16)	3.46 (204.45)	3.45 (2.97)	1.79 (1.52)
∗Taste disorder	116	18.14 (15.11–21.79)	18.05 (1856.05)	17.93 (15.39)	4.16 (3.9)
∗Diabetic ketoacidosis	105	12.34 (10.19–14.96)	12.29 (1084.27)	12.24 (10.42)	3.61 (3.33)
∗Dysphagia	86	2.43 (1.97–3)	2.42 (71.97)	2.42 (2.03)	1.28 (0.97)
Blood creatinine increased	84	3.54 (2.85–4.38)	3.53 (152.1)	3.52 (2.95)	1.82 (1.5)
∗Feeding disorder	79	12.16 (9.74–15.17)	12.12 (802.4)	12.07 (10.03)	3.59 (3.27)
Blood glucose abnormal	79	11.98 (9.6–14.95)	11.94 (788.77)	11.89 (9.88)	3.57 (3.25)
Neoplasm malignant	75	3.16 (2.52–3.96)	3.15 (109.99)	3.15 (2.6)	1.65 (1.32)
∗Eating disorder	75	9.79 (7.8–12.29)	9.76 (587.62)	9.73 (8.04)	3.28 (2.95)
Metastases to liver	71	11.63 (9.21–14.69)	11.6 (684.48)	11.55 (9.5)	3.53 (3.19)
Breast cancer metastatic	69	18.27 (14.42–23.17)	18.22 (1115.33)	18.1 (14.84)	4.18 (3.83)
∗Thirst	66	10.56 (8.29–13.45)	10.53 (567.08)	10.49 (8.57)	3.39 (3.04)
Tumor marker increased	61	35.61 (27.65–45.86)	35.51 (2018.22)	35.04 (28.36)	5.13 (4.76)
Rash pruritic	55	2.93 (2.25–3.82)	2.93 (69.8)	2.93 (2.34)	1.55 (1.16)
∗Lip swelling	49	4.15 (3.13–5.49)	4.14 (116.64)	4.14 (3.27)	2.05 (1.64)
Metastases to bone	43	7.65 (5.67–10.32)	7.64 (247.39)	7.62 (5.93)	2.93 (2.49)
∗Ageusia	42	4.69 (3.46–6.35)	4.68 (121.53)	4.68 (3.63)	2.23 (1.78)
∗Pollakiuria	42	2.84 (2.1–3.85)	2.84 (50.08)	2.84 (2.2)	1.51 (1.06)
∗Mucosal inflammation	40	4.34 (3.18–5.92)	4.33 (102.39)	4.33 (3.34)	2.11 (1.66)
∗Pneumonitis	39	4.18 (3.05–5.73)	4.18 (94.12)	4.17 (3.21)	2.06 (1.6)
∗Ketoacidosis	34	12.78 (9.12–17.9)	12.76 (366.75)	12.7 (9.58)	3.67 (3.18)
∗Frequent bowel movements	28	3.05 (2.1–4.41)	3.04 (38.38)	3.04 (2.23)	1.6 (1.07)
Liver function test increased	27	3.74 (2.56–5.46)	3.74 (54.09)	3.73 (2.72)	1.9 (1.35)
Glycosylated hemoglobin increased	27	3.06 (2.1–4.46)	3.06 (37.34)	3.05 (2.23)	1.61 (1.07)
∗Stevens‐Johnson syndrome	25	3.1 (2.09–4.59)	3.1 (35.49)	3.1 (2.23)	1.63 (1.06)
∗Oral mucosal blistering	25	10.92 (7.37–16.18)	10.91 (224.17)	10.87 (7.82)	3.44 (2.87)
∗Retching	24	3.19 (2.14–4.76)	3.18 (35.93)	3.18 (2.28)	1.67 (1.09)
Skin reaction	24	4.88 (3.27–7.28)	4.87 (73.79)	4.87 (3.48)	2.28 (1.71)
∗Glossodynia	24	3.06 (2.05–4.57)	3.06 (33.26)	3.06 (2.19)	1.61 (1.03)
∗Hyperglycemic hyperosmolar nonketotic syndrome	23	55.01 (36.39–83.15)	54.95 (1192.83)	53.82 (38.09)	5.75 (5.15)
Blood glucose fluctuation	23	5.79 (3.85–8.72)	5.79 (90.86)	5.77 (4.1)	2.53 (1.94)
Product availability issue	23	3.19 (2.12–4.8)	3.19 (34.49)	3.18 (2.26)	1.67 (1.08)
Tumor marker decreased	23	429.3 (276.03–667.66)	428.85 (8415.16)	367.73 (254.12)	8.52 (7.89)
Metastases to central nervous system	22	4.99 (3.29–7.59)	4.99 (70.05)	4.98 (3.51)	2.32 (1.71)
∗Blood sodium decreased	22	3.25 (2.14–4.94)	3.25 (34.28)	3.25 (2.29)	1.7 (1.1)
Therapy change	20	6 (3.87–9.3)	5.99 (83.03)	5.98 (4.14)	2.58 (1.95)
Metastases to lung	19	4.72 (3.01–7.4)	4.72 (55.55)	4.71 (3.23)	2.24 (1.59)
Aphthous ulcer	19	5.38 (3.43–8.45)	5.38 (67.61)	5.37 (3.68)	2.43 (1.78)
Metastasis	17	6.67 (4.14–10.74)	6.67 (81.69)	6.65 (4.47)	2.73 (2.05)
Product supply issue	17	7.18 (4.46–11.56)	7.18 (90.11)	7.16 (4.81)	2.84 (2.16)
Hospice care	16	4.72 (2.89–7.71)	4.71 (46.75)	4.71 (3.12)	2.24 (1.53)
Skin toxicity	15	8.19 (4.93–13.6)	8.19 (94.33)	8.16 (5.34)	3.03 (2.31)
Electrolyte imbalance	15	3.76 (2.26–6.23)	3.75 (30.26)	3.75 (2.45)	1.91 (1.18)
∗Mouth swelling	14	6.93 (4.1–11.71)	6.93 (70.81)	6.91 (4.45)	2.79 (2.04)
∗Polydipsia	14	9.72 (5.75‐16.44)	9.72 (109.08)	9.68 (6.24)	3.28 (2.53)
∗Feces soft	14	5.76 (3.41–9.73)	5.75 (54.88)	5.74 (3.7)	2.52 (1.78)
∗Pharyngeal swelling	13	4.15 (2.41–7.15)	4.15 (31.03)	4.14 (2.63)	2.05 (1.28)
∗Polyuria	13	4.4 (2.56–7.59)	4.4 (34.12)	4.4 (2.79)	2.14 (1.36)
Neoplasm	13	3.19 (1.85–5.5)	3.19 (19.52)	3.19 (2.02)	1.67 (0.9)
Oral disorder	13	4.62 (2.68–7.95)	4.61 (36.73)	4.61 (2.92)	2.2 (1.43)
Second primary malignancy	13	4.24 (2.46–7.3)	4.23 (32.07)	4.23 (2.68)	2.08 (1.31)
∗Onychoclasis	13	5.31 (3.08–9.15)	5.3 (45.32)	5.3 (3.36)	2.4 (1.63)
∗Lip blister	12	12.28 (6.97–21.66)	12.28 (123.72)	12.22 (7.6)	3.61 (2.81)
∗Gingival pain	12	4.22 (2.39–7.43)	4.22 (29.41)	4.21 (2.62)	2.07 (1.27)
Lymphoedema	12	4.95 (2.81–8.72)	4.95 (37.72)	4.94 (3.07)	2.3 (1.5)
∗Cheilitis	11	5.61 (3.1–10.14)	5.61 (41.55)	5.6 (3.41)	2.48 (1.65)
∗Hepatic lesion	10	6.57 (3.53–12.23)	6.57 (47.13)	6.56 (3.9)	2.71 (1.84)
∗Tongue discomfort	10	6.8 (3.66–12.66)	6.8 (49.36)	6.79 (4.04)	2.76 (1.89)
∗Lip dry	10	3.4 (1.83–6.32)	3.4 (16.88)	3.39 (2.02)	1.76 (0.89)
∗Oral mucosal eruption	9	16.65 (8.65–32.08)	16.65 (131.53)	16.55 (9.56)	4.05 (3.13)
Blood creatine increased	9	5.65 (2.94–10.87)	5.65 (34.37)	5.64 (3.26)	2.5 (1.58)
Carcinoembryonic antigen increased	9	13.23 (6.87–25.47)	13.23 (101.19)	13.16 (7.61)	3.72 (2.8)
Product distribution issue	9	3.86 (2.01–7.42)	3.86 (19.03)	3.85 (2.23)	1.95 (1.03)
Metastases to spine	9	7.78 (4.04–14.96)	7.77 (52.96)	7.75 (4.48)	2.95 (2.04)
Breast cancer stage iv	8	7.44 (3.71–14.89)	7.43 (44.42)	7.42 (4.15)	2.89 (1.93)
Bone cancer	8	4.53 (2.26–9.07)	4.53 (21.98)	4.52 (2.53)	2.18 (1.21)
∗Diabetic coma	8	7.63 (3.81–15.27)	7.62 (45.91)	7.6 (4.25)	2.93 (1.96)
Renal function test abnormal	7	4.37 (2.08–9.18)	4.37 (18.16)	4.36 (2.35)	2.13 (1.1)
Intra‐abdominal fluid collection	7	8.94 (4.25–18.77)	8.93 (49.16)	8.91 (4.79)	3.15 (2.13)
Drug tolerance	7	3.84 (1.83–8.07)	3.84 (14.71)	3.84 (2.06)	1.94 (0.92)
Mucosal dryness	7	9.81 (4.67–20.62)	9.81 (55.18)	9.78 (5.25)	3.29 (2.27)
∗Red cell distribution width increased	6	4.04 (1.81–9)	4.04 (13.71)	4.04 (2.07)	2.01 (0.92)
∗Urine output increased	6	6.25 (2.8–13.93)	6.25 (26.39)	6.24 (3.19)	2.64 (1.55)
∗Nail discoloration	6	4.29 (1.93–9.56)	4.29 (15.12)	4.29 (2.19)	2.1 (1.01)
Cancer pain	6	6.32 (2.84–14.09)	6.32 (26.82)	6.31 (3.23)	2.66 (1.57)
∗Near death experience	6	5.63 (2.52–12.53)	5.62 (22.76)	5.61 (2.87)	2.49 (1.4)
∗Insulin resistance	6	8.35 (3.75–18.62)	8.35 (38.69)	8.33 (4.26)	3.06 (1.96)
Hormone receptor positive HER2‐breast cancer	6	76.45 (33.94–172.21)	76.43 (433.77)	74.25 (37.64)	6.21 (5.11)
Hepatic neoplasm	5	4.25 (1.77–10.22)	4.25 (12.41)	4.24 (2.04)	2.09 (0.91)
∗Oral mucosal erythema	5	9.11 (3.78–21.92)	9.11 (35.95)	9.08 (4.35)	3.18 (2)
∗Lip pain	5	4.88 (2.03–11.74)	4.88 (15.4)	4.87 (2.34)	2.28 (1.11)
Product packaging difficult to open	5	6.73 (2.8–16.2)	6.73 (24.34)	6.72 (3.22)	2.75 (1.57)
∗Salivary hyposecretion	5	13.88 (5.76–33.43)	13.88 (59.43)	13.81 (6.62)	3.79 (2.61)
Food aversion	5	9.15 (3.8–22.03)	9.15 (36.17)	9.12 (4.37)	3.19 (2.01)
∗Diabetic metabolic decompensation	5	10.12 (4.21–24.37)	10.12 (40.95)	10.09 (4.84)	3.33 (2.15)
Pik3ca‐activated mutation	5	45.15 (18.65–109.33)	45.14 (212.1)	44.38 (21.18)	5.47 (4.28)
∗Lip exfoliation	4	8.44 (3.16–22.52)	8.44 (26.13)	8.41 (3.7)	3.07 (1.78)
Tumour marker abnormal	4	33.97 (12.67–91.12)	33.97 (126.32)	33.54 (14.69)	5.07 (3.77)
∗Carbohydrate antigen increased	4	14.9 (5.57–39.81)	14.9 (51.55)	14.82 (6.51)	3.89 (2.59)
∗Noninfective gingivitis	4	7.24 (2.71–19.32)	7.24 (21.44)	7.22 (3.18)	2.85 (1.56)
∗Mucosal disorder	4	4.63 (1.73–12.34)	4.63 (11.35)	4.62 (2.03)	2.21 (0.92)
∗Hepatic mass	4	5.98 (2.24–15.96)	5.98 (16.55)	5.97 (2.63)	2.58 (1.29)
∗Dysentery	4	6 (2.25–15.99)	5.99 (16.61)	5.98 (2.63)	2.58 (1.29)
Gene mutation	4	5.74 (2.15–15.32)	5.74 (15.62)	5.73 (2.52)	2.52 (1.23)
∗Paracentesis	4	17.75 (6.64–47.45)	17.75 (62.78)	17.63 (7.74)	4.14 (2.84)
Creatinine renal clearance increased	4	14.9 (5.57–39.81)	14.9 (51.55)	14.82 (6.51)	3.89 (2.59)
∗Discharge	4	8.79 (3.29–23.46)	8.79 (27.52)	8.76 (3.85)	3.13 (1.84)
Metastases to meninges	4	5.2 (1.95–13.88)	5.2 (13.56)	5.19 (2.29)	2.38 (1.08)
Hematological infection	4	6.69 (2.51–17.86)	6.69 (19.32)	6.68 (2.94)	2.74 (1.45)
∗Colonic abscess	4	12.72 (4.76–33.99)	12.72 (42.99)	12.66 (5.57)	3.66 (2.37)
Mean cell hemoglobin increased	3	5.2 (1.67–16.13)	5.19 (10.14)	5.19 (2.01)	2.37 (0.93)
∗Gingival discomfort	3	13.33 (4.29–41.47)	13.33 (34.05)	13.27 (5.13)	3.73 (2.28)
∗Solar lentigo	3	8.84 (2.85–27.48)	8.84 (20.8)	8.82 (3.41)	3.14 (1.69)
∗Blood electrolytes abnormal	3	6.69 (2.15–20.78)	6.69 (14.48)	6.67 (2.59)	2.74 (1.29)
∗Tongue erythema	3	10.78 (3.47–33.51)	10.78 (26.51)	10.74 (4.16)	3.42 (1.98)
Carbohydrate antigen increased	3	53.61 (17.09–168.21)	53.61 (151.72)	52.53 (20.18)	5.72 (4.25)
∗Fungating wound	3	160.84 (50.09–516.42)	160.82 (448.45)	151.42 (57.05)	7.24 (5.75)
∗Oral mucosal roughening	3	36.94 (11.82–115.48)	36.93 (103.4)	36.43 (14.04)	5.19 (3.73)
Creatinine renal clearance abnormal	3	17.47 (5.61–54.37)	17.46 (46.25)	17.35 (6.71)	4.12 (2.67)
∗Gingival erythema	3	9.51 (3.06–29.54)	9.51 (22.75)	9.48 (3.67)	3.24 (1.8)
∗Product blister packaging issue	3	8.34 (2.68–25.9)	8.34 (19.31)	8.31 (3.22)	3.06 (1.61)
∗Nutritional condition abnormal	3	12.2 (3.92–37.92)	12.19 (30.69)	12.14 (4.7)	3.6 (2.15)
Metastases to bone marrow	3	16.78 (5.39–52.23)	16.78 (44.23)	16.68 (6.45)	4.06 (2.61)
∗Anisocytosis	3	12.89 (4.14–40.08)	12.89 (32.73)	12.83 (4.96)	3.68 (2.23)
∗QRS axis abnormal	3	8.86 (2.85–27.54)	8.86 (20.85)	8.84 (3.42)	3.14 (1.7)

*Note:* ∗ indicates positive signals not mentioned in the package insert.

Abbreviations: 95% CI, two‐sided for ROR; CI, confidence interval; EBGM, the empirical Bayes geometric mean; EBGM05 and IC025 lower one‐sided for EBGM and IC; PRR, proportional reporting ratio; ROR, reporting odds ratio; *χ* ^2^, chi‐squared.

In terms of signal strength, ranking by ROR values revealed that the top five AEs with the highest signal intensity were hyperglycemia (ROR 97.63, PRR 92.53), hyperglycemic hyperosmolar nonketotic syndrome (ROR 55.01, PRR 54.95), taste disorder (ROR 18.14, PRR 18.05), oral pain (ROR 14.73, PRR 14.65), and stomatitis (ROR 14.19, PRR 14.01). Among the 133 positive signals, some positive AE signals were not mentioned in the package insert, including diabetic ketoacidosis (DKA), pneumonitis, Stevens–Johnson syndrome, and hyperglycemic hyperosmolar nonketotic syndrome, et al.

### 3.4. Integrated Analysis of Potential Toxic Targets of Capivasertib and Key SOCs

**Figure 3:**
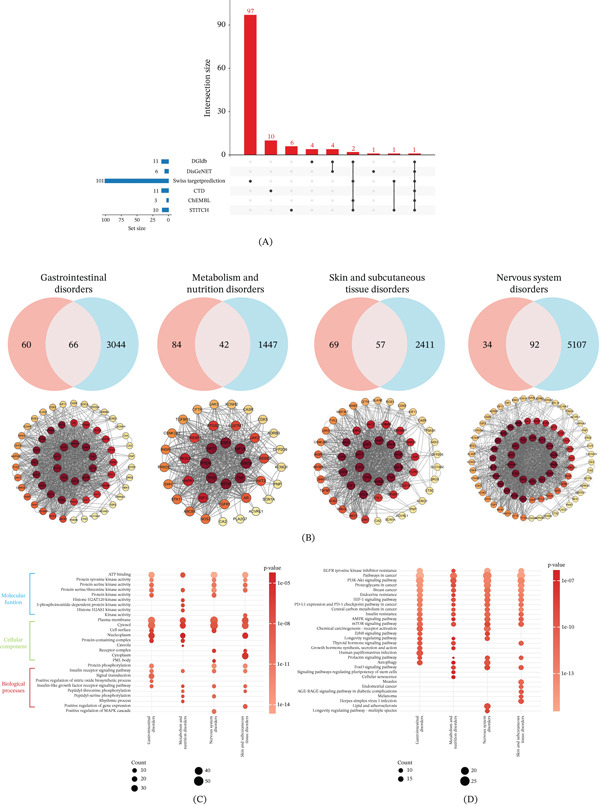
Integrated analysis of potential toxic targets of capivasertib and key SOCs. (A) Sources of predicted targets of capivasertib. (B) Venn diagram showing the overlap between predicted targets and key SOCs of capivasertib, along with the PPI network map of common targets shared by capivasertib and the four key SOCs. (C) GO functional enrichment analysis of potential toxic targets of the four key SOCs. (D) KEGG pathway enrichment analysis of potential toxic targets of the four key SOCs.

To elucidate the potential toxicological mechanisms underlying capivasertib‐associated AEs, we focused on key SOCs with high reporting frequencies (> 3 AEs), excluding nonspecific categories such as “general disorders and administration site conditions” and “investigations.” A total of 126 predicted molecular targets of capivasertib were retrieved from seven databases (Figure 3A). By intersecting these targets with disease‐specific genes from the GeneCards database, we identified the overlapping genes as potential toxicity targets for the four key SOCs (Figure 3B).

These intersecting targets were subsequently used to construct a PPI network via the STRING database and visualized in Cytoscape. Network topology analysis revealed comparable central nodes across all four SOC categories, implying that capivasertib‐induced toxicities may be driven by common fundamental pathways.

To further investigate the biological roles of these shared targets, GO and KEGG enrichment analyses were performed using the DAVID database. The GO analysis highlighted protein phosphorylation, insulin and insulin‐like growth factor receptor signaling pathways, general signal transduction, and the positive regulation of nitric oxide biosynthesis as the top significantly enriched biological processes (Figure 3C). Concordantly, KEGG enrichment revealed that the top 20 pathways—including PI3K‐Akt signaling, EGFR tyrosine kinase inhibitor resistance, endocrine resistance, and the HIF‐1 signaling pathway—were largely conserved across the four SOCs (Figure 3D). Collectively, these findings indicate substantial cross‐regulation and the involvement of shared signaling cascades in mediating the diverse adverse effects of capivasertib.

### 3.5. Molecular Interaction Patterns Between Capivasertib and Key Toxicity Targets: Docking and Dynamics Simulations

**Figure 4:**
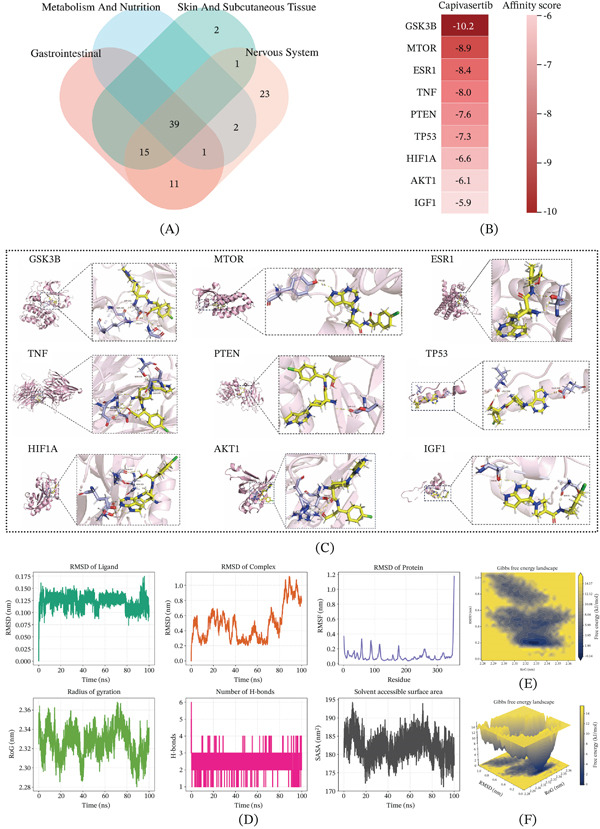
Molecular interaction patterns between capivasertib and key toxicity targets: docking and dynamics simulations. (A) Venn diagram of common targets of capivasertib in four key SOCs. (B) Heat map of binding energy between capivasertib and proteins expressed by key toxicity genes. (C) Binding site of capivasertib to proteins expressed by key toxicity genes. (D) Molecular dynamics simulation of the capivasertib–GSK3B complex over 100 ns. Panels show (from left to right, top to bottom) RMSD of the ligand, RMSD of the protein–ligand complex, RMSF of protein residues, radius of gyration (RoG), number of hydrogen bonds, and solvent‐accessible surface area (SASA). Converged RMSD and RoG profiles, together with persistent intermolecular hydrogen bonds and stable SASA, confirm the formation of a robust protein–ligand complex. (E) Two‐dimensional and (F) three‐dimensional Gibbs free energy landscapes projected onto RMSD and RoG coordinates, revealing a pronounced global energy minimum indicative of a thermodynamically favorable and stable bound conformation.

To identify the core toxicity targets across the four key SOCs, we analyzed a subset of 39 overlapping genes (Figure 4A). Cytoscape‐based network topology analysis revealed nine hub proteins with the highest degree centrality: AKT1, IGF1, GSK3B, HIF1A, TNF, ESR1, MTOR, PTEN, and TP53. Highly enriched in key KEGG pathways, these proteins emerge as potential primary mediators of capivasertib‐induced adverse effects. To validate these findings, computational molecular docking was performed to assess the interactions between capivasertib and these hub targets. Capivasertib exhibited strong binding affinities across all targets, yielding binding free energies of ≤ −5 kcal/mol (Figure 4B,C). These thermodynamically favorable interactions strongly corroborate our network toxicology predictions, suggesting that direct binding to these proteins significantly contributes to the off‐target toxicological mechanisms of capivasertib.

MD simulations were performed to evaluate the binding stability of capivasertib to GSK3B over a 100 ns trajectory (Figure 4D). The RMSD profiles of both the ligand and the protein‐ligand complex exhibited stable fluctuations with convergent trajectories, indicating that the complex reached equilibrium shortly after the initial simulation phase and remained structurally stable throughout the production run. The RoG displayed consistent values with minor fluctuations, suggesting maintenance of the overall compactness and structural integrity of the complex. Analysis of hydrogen bonding interactions revealed persistent and reproducible H‐bond patterns, further supporting the formation of a stable binding interface. Additionally, the SASA remained relatively constant, indicating minimal conformational exposure of the binding pocket to the solvent environment. Gibbs FEL analysis in both 2D and 3D representations identified a well‐defined global energy minimum, corresponding to the most favorable conformational state of the capivasertib‐GSK3B complex (Figure 4E,F). Collectively, these MD data demonstrate that capivasertib forms a thermodynamically stable and conformationally constrained complex with GSK3B, corroborating its strong binding affinity identified in the docking analysis.

### 3.6. Single‐Cell Landscape of GSK3B Heterogeneity and Microenvironmental Crosstalk in BC

**Figure 5:**
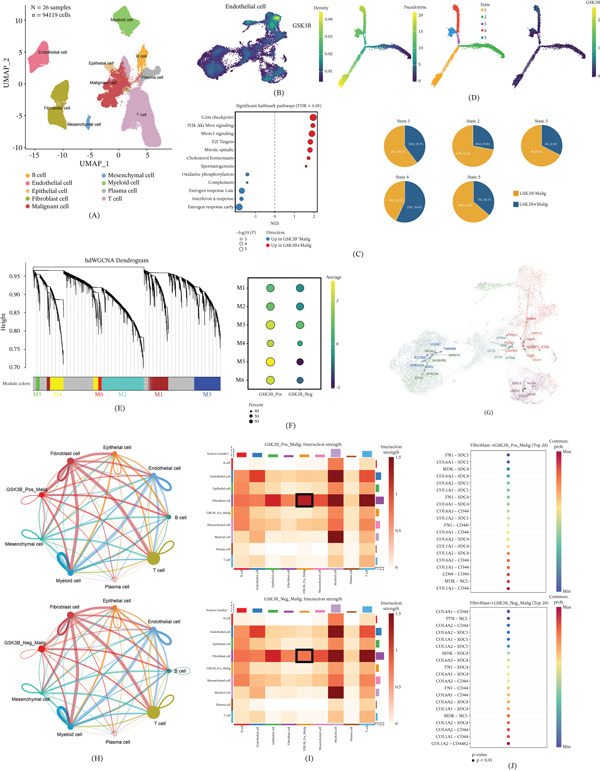
Comprehensive single‐cell transcriptomic analysis of GSK3B expression, pseudotime trajectory, coexpression network, and cell–cell communication in breast cancer. (A) UMAP visualization of 94,119 cells from 26 breast cancer samples, identifying nine major cell types. (B) Density plots of GSK3B expression on the UMAP embedding in the epithelial cells. (C) GSEA of significant Hallmark pathways (FDR < 0.05) between GSK3B‐positive and GSK3B‐negative malignant cells. Dot size represents −log_10_(*P*); red and blue indicate pathways upregulated in GSK3B_pos_Malig and GSK3B_neg_Malig, respectively. (D) Monocle pseudotime trajectory analysis of malignant cells. Top row: cells colored by pseudotime (left), state (middle), and GSK3B expression (right). Bottom row: pie charts showing the proportion and counts of GSK3B^+^ Malig (orange) and GSK3B^−^ Malig (blue) within each state (*p* < 0.0001). (E) hdWGCNA dendrogram and module assignment (M1–M6). (F) Dot plot of module hub gene expression in GSK3B‐positive versus GSK3B‐negative groups. Dot size indicates percent expressed; color indicates average expression. (G) UMAP network visualization of module genes. (H) CellChat‐inferred cell‐cell communication networks for GSK3B_Pos_Malig (top) and GSK3B_Neg_Malig (bottom), with line thickness representing interaction strength. (I) Heatmaps of pairwise interaction strength among cell types for GSK3B_Pos_Malig (top) and GSK3B_Neg_Malig (bottom). The color scale represents interaction probability, ranging from low (blue) to high (red). (J) Top 20 significant ligand‐receptor interactions from fibroblasts to GSK3B_Pos_Malig (top, *p* < 0.01) and GSK3B_Neg_Malig (bottom, *p* < 0.01). Dot size represents *p* value; color represents communication probability.

To investigate the single‐cell landscape of GSK3B—the top target exhibiting the strongest binding affinity to capivasertib—we performed comprehensive transcriptomic profiling of 94,119 cells derived from 26 BC samples. Unsupervised clustering identified nine major cell types, including B cells, endothelial cells, epithelial cells, fibroblasts, malignant cells, mesenchymal cells, myeloid cells, plasma cells, and T cells (Figure 5A). Density plotting of GSK3B expression within the epithelial compartment revealed distinct subpopulations with elevated GSK3B levels, suggesting heterogeneous expression of this therapeutic target among tumor epithelial cells (Figure 5B). To delineate the biological consequences of GSK3B expression in malignant cells, we stratified malignant epithelial cells into GSK3B^+^ Malig and GSK3B^−^ Malig subsets and performed GSEA using Hallmark pathways. GSK3B^+^ malignant cells displayed significant enrichment of proliferation‐related and oncogenic signaling pathways, including G2‐M checkpoint, PI3K‐AKT‐mTOR signaling, mTORC1 signaling, E2F targets, MYC targets, and mitotic spindle pathways (Figure 5C). Conversely, GSK3B^−^ malignant cells were characterized by upregulation of estrogen response pathways (both early and late), indicating that GSK3B expression defines a transcriptionally distinct aggressive subtype with enhanced proliferative capacity and reduced hormone dependency. Pseudotime trajectory analysis using Monocle 2 reconstructed the developmental ordering of malignant cells across five discrete states (States 1–5). Cells were distributed along a continuous trajectory with clear branch structure, and GSK3B expression was dynamically regulated across pseudotime (Figure 5D, top). Notably, the proportional representation of GSK3B^+^ and GSK3B^−^ malignant cells differed significantly among states (*p* < 0.0001), with certain states predominantly occupied by GSK3B‐high cells, suggesting that GSK3B expression is associated with specific stages of tumor progression or differentiation (Figure 5D, bottom).

To identify GSK3B‐associated transcriptional programs, we conducted hdWGCNA and identified six distinct coexpression modules (M1–M6) (Figure 5E). Module hub gene expression profiling revealed divergent patterns between GSK3B^+^ and GSK3B^−^ groups: specific modules (e.g., M1, M2, M6) exhibited higher average expression and greater cell proportions in one group versus the other, indicating group‐specific modular transcriptional signatures (Figure 5F). UMAP‐based network visualization further illustrates the modular organization and interconnectivity of hub genes within these co‐expression networks (Figure 5G).

Finally, we interrogated cell‐cell communication landscapes using CellChat to compare how GSK3B^+^ and GSK3B^−^ malignant cells interact with the tumor microenvironment. The overall communication networks revealed distinct interaction topologies and differential interaction strengths between the two groups (Figure 5H). Pairwise interaction strength heatmaps demonstrated that GSK3B status profoundly reshaped intercellular communication patterns among malignant cells and stromal/immune compartments (Figure 5I). Specifically, fibroblast‐to‐malignant cell signaling was markedly divergent: the top 20 significant ligand‐receptor pairs (*p* < 0.01) showed that GSK3B^+^ malignant cells preferentially received signals through interactions such as FN1–SDC1, COL4A1–SDC1, MDK–SDC1, and COL4A1–SDC4, whereas GSK3B^−^ malignant cells exhibited dominant communication via COL4A1–CD44, PTN–NCL, and alternative FN1‐mediated axes (Figure 5J). These findings collectively demonstrate that GSK3B expression not only defines the transcriptional and proliferative identity of BC cells but also remodels their communicative crosstalk with the tumor microenvironment, providing a mechanistic basis for targeting GSK3B with capivasertib in a cell‐context‐dependent manner.

### 3.7. Landscape of PIK3CA/AKT/PTEN Alterations in BC

**Figure 6:**
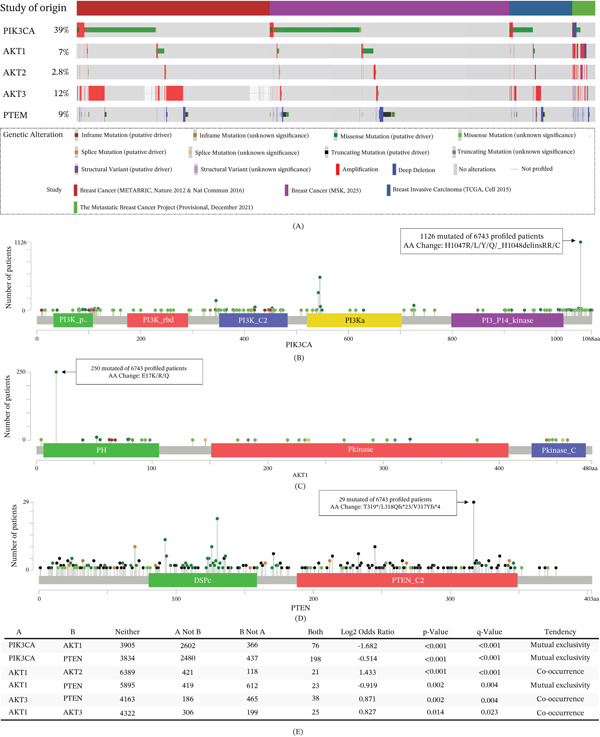
Genomic Landscape and Interplay of the PI3K/AKT/PTEN Pathway in Breast Cancer. (A) OncoPrint visualization of genetic alterations in *PIK3CA, AKT1, AKT2, AKT3,* and *PTEN* across 6743 patients from four major cohorts (METABRIC, MSK, TCGA, and the metastatic breast cancer project). (B–D) Lollipop plots illustrating the distribution and frequency of somatic mutations across the protein domains of (B) PIK3CA, (C) AKT1, and (D) PTEN. (E) Statistical analysis of gene‐pair associations demonstrating mutual exclusivity and co‐occurrence.

Capivasertib is an Akt potent inhibitor working in the PI3K/Akt pathway; as Akt activity is negative‐regulated by PTEN, therefore, we checked whether there were some relations to PIK3CA/AKT and PTEN gene mutation and its frequency. In general, 6743 BC patients from four large‐scale WES projects: METABRIC project, MSK project, TCGA‐Breast Cancer Project, and Metastatic Breast Cancer Project. PIK3CA mutations were also observed commonly (39%) and the majority of these were hotspot missense substitutions (notably H1047R/L/Y/Q in 1,126/6,743 patients, with secondary helical‐domain hotspots E542K/E545K, as shown in Figure 6B), especially for metastatic tumors (Figure 6A), where we saw Akt1 mutations were predominantly amino acid changes whereas for the metastatic tumors, copy number gain was predominant suggesting that perhaps capivasertib may be more effective in treating metastatic disease. For Akt3, the primary change was an amplification (12%); whereas PTEN alterations were about 9% of cases. We found some kind of genetic alterations, most frequently deletion type changes (Figure 6A) and the lollipop plots for PI3KCA, AKT1, and PTEN (Figure 6B–D), indicating certain mutation hot spots which are potentially more therapeutically relevant mutations to benefit from the treatment with capivasertib. Furthermore, we observed that of all the 6743 BCs Figure 6: E. PIK3CA, AKT1, and PTEN are mutually exclusive suggesting they play a major role as driver events for this disease which is promising when it comes to using capivasertib to treat BCs bearing these specific mutations.

## 4. Discussion

In this study, we provided a first‐in‐depth assessment for AEs of capivasertib using observational (real world) drug safety data available in FDA Adverse Event Reporting System (FAERS), and by applying predictive modeling approaches that rely on computational toxicology studies. We detected via our FAERS disproportionality analysis the following safety signals: with digestive diseases (ROR = 2.60), as well as, for metabolic disorders (ROR = 5.74). The most important abnormality was hyperglycemia, consistent with the mechanism of action of this drug to inhibit Akt within the insulin pathway. This is also consistent with safety findings from trials and package insert descriptions [17, 18].

PI3K‐AKT pathway mutations have been found in most patients with BC and play an important role in tumor proliferation and chemoresistance. Approximately 40% of hormone receptor‐positive, HR+/HER2− BC harbor PIK3CA activating mutations, which constitutively activate downstream AKT signaling and enhance tumor cell survival and growth [19]. In addition, AKT1 hotspot mutations (particularly E17K) and loss‐of‐function alterations in the PTEN tumor suppressor gene—detected in 5%–10% of HR+ tumors and more commonly in triple‐negative BC—similarly drive aberrant AKT pathway activation [20, 21]. As a pivotal serine/threonine kinase in the PI3K/AKT/mTOR axis, AKT represents a compelling therapeutic target in pathway‐altered BC [22].

Capivasertib (AZD5363) is a potent, selective ATP‐competitive inhibitor of all three AKT isoforms (AKT1/2/3), specifically developed to counteract hyperactivation of the PI3K–AKT–mTOR pathway in BC [2]. Clinical investigations, including the Phase III CAPItello‐291 trial, have demonstrated its efficacy in biomarker‐enriched populations with alterations in PIK3CA, AKT1, or PTEN [23]. Notably, capivasertib has exhibited substantial antitumor activity as a monotherapy in patients with AKT1^E17K^ mutations, supporting its utility in genomically defined BC subtypes [24].

FAERS safety results were consistent with known adverse events reported for capivasertib within its product label that has been cleared by FDA (e.g., skin‐related adverse events), diarrhea; hyperglycemia; stomatitis; emesis; fatigue. There are other serious side effects not sufficiently reported in the literature that we have discovered during this study: changes in the mouth lining, pneumonia, and a serious skin disease called Stevens–Johnson syndrome. All of these effects may be attributed to the drug′s action as an inhibitor of the AKT signaling cascade, which disrupts biological processes controlling metabolism, tissue barrier function and immune system activity. All these findings fit well in line with other clinical evidences including others by Rugo et al. (2024), for instance, on the GI adverse effects, skin toxicities, and hyperglycemia as the main Grade 3 or higher adverse events seen with capivasertib treatment, is often rapid, after starting therapy, because it interrupts the PI3K–AKT pathway [18]. In this study, we confirmed the presence of gastrointestinal issues as well as metabolic and nutritional disorders among the most frequent observations. The highest measures of disproportionality were found within this SOC that validated known safety signals and indicated potential new safety concerns.

The package insert for capivasertib lists the following AEs: diarrhea, nausea, vomiting, skin rash, hyperglycemia, weakness, stomatitis, and laboratory abnormalities including decreased lymphocytes and increased creatinine level. The data from the FAERS database corroborated that these safety signals and diarrhea were the most common serious adverse event, as reflected in strong values of disproportionality metrics (ROR and IC). Such findings also hold for the CAPItello‐291 clinical trial, where about 72% of the participants experienced diarrhea (any grade) and about 9.3% had severe diarrhea (Grade 3 or more) [18]. Biologically speaking, the AKT pathway plays an important role in maintaining epithelial cell integrity and functionality. Inhibition of this pathway disrupts electrolyte transport and epithelial barrier function in the gut, increased risk of osmotic secretory diarrhea. Skin‐related AE was a commonly reported AE in the FAERS database, including rash, pruritus, dermatologic reactions (hand–foot), and individual cases of serious cutaneous adverse reaction such as Stevens–Johnson syndrome. These skin phenotypes are consistent with expected drug effects resulting from inhibition of EGFR/PI3K‐AKT pathway, likely to be due to inflammation induced via loss of viability signaling pathways within the epidermis and keratinocytes [25, 26]. The above findings confirm the recommendation for close monitoring, as well as potential dose reduction in case of serious skin reactions from prescribing guidelines: validating the biological plausibility for toxicities associated with AKT pathway inhibition.

Hyperglycemia was noted to be a commonly reported ADR, as well as an important FAERs signal (*n* = 976; ROR 14.87, PRR 14.25). The AKT protein is important for the signal transduction of insulin signaling. Capivasertib inhibition of the pathway disrupts those processes, which leads to increased blood glucose concentrations. In the CAPItello study, diarrhea, rash, and hyperglycemia have shown to be directly related to the gut microbiota and hyperglycemia to PI3K/AKT inhibitors [18]. Likewise, in SOLAR‐1 study, hyperglycemia was also identified as one of the most common side effects with capivasertib, (64%) overall (any degree), and 37% had Grade 3 or greater events [27]. Not only our pharmacovigilance study showed the occurrence of mild glucose metabolism disorders, it could show serious metabolic disorders as well, including DKA (ROR 12.34, PRR 12.29), and hyperosmolar hyperglycemic state (HHS; ROR 55.01, PRR 54.95). Because other author reporting hyperglycemia on nondiabetic patient under treatment by capivasertib [28]. The incidence is low according clinical trial (~0, 3%), they underline that monitoring of glucose levels is essential [29]. From another point of view (biological) inactivation of AKT will eliminate the negative phosphorylation effect on FoxO1 and GSK‐3*β* that leads to activation of gluconeogenesis and glycogenolysis [30, 31]. Furthermore, compensatory up‐regulation of insulin receptor and IGF1R signaling after inhibition of AKT, leading to greater insulin resistance and possibly higher glucose levels [32]. The expected pharmacodynamic effects thus suggest that routine monitoring of fasting blood sugar levels, as well as the prudent use of hypoglycemic agents in patients on therapy with capivasertib may be warranted.

Our disproportionality analysis identified an AE already described (ROR 14.19, PRR 14.01), which is highly related to the drug′s mechanism of action. The oral mucosa and gastrointestinal tract are composed of epithelial cells, the proliferation of which depends on PI3K/AKT signaling. Inhibiting of this pathway—such as through AKT or mTOR inhibitors—often results in inflammatory conditions of the mucosa, such as oral ulcers [33]. In the clinical trial for CAPItello‐291, 14.6% of patients developed stomatitis and 2.0% had Grade 3 or higher toxicity [18]. From FAERS reports, we also found symptoms such as mouth pain, erythematous gums; inflammation of the mucosa; and disturbance of taste sensation (loss or impairment). It is possible that these symptoms represent milder, or different manifestations of mucosal injury associated with the PI3K/AKT pathway inhibition. Xerostomia and dysgeusia are likely to reflect dysfunction of the salivary glands, or minimal mucositis, whereas edema of lips, pharynx could be signs for a local vasculitis or hypersensitivity reaction. Notably, capivasertib may cause serious skin toxicities such as erythema multiforme, or generalized hypersensitivity with eosinophilia. Little data exists on the frequency of a particular oral toxicity associated with the use of capivasertib but include similar PI3K/AKT/mTOR pathway inhibitors which showed significant oral toxicity requiring prophylaxis. The use of local steroids or dexamethasone‐containing mouthwashes, which are often used to treat mTOR inhibitor‐induced stomatitis may be helpful in this regard [34, 35].

In addition to confirming some well‐known safety signals, our review identified additional safety issues with capivasertib that were underappreciated in pre‐market development. Chief among these was hypoglycemia as an important safety signal. Besides DKA or HHS, we found a surprising large number of patients with dehydration, weight loss, polydipsia, polyuria, and electrolyte disturbances are signs of the development of diabetic ketoacidosis due to high levels of glucose in blood that is not controlled. Such symptoms are consistent with the sequelae of osmotic diuresis secondary to glucosuria, and which causes large fluid and electrolyte losses which may result in dehydration and renal failure. The observed incidence in decreased body weight and difficulty eating may relate to the appetite suppressive effects of capivasertib and cachexia, which is in line with what has been observed using other PI3K/AKT pathway inhibitors, likely as a consequence of perturbed insulin/IGF‐1 signaling and poor nutrient uptake; these results underline that careful monitoring of metabolism should be undertaken when administering this agent. Testing of blood sugars before and after meals is suggested in patients who receive capivasertib, and assessments of renal function and hydration status. Prompt initiation of antihyperglycemic therapy, such as insulin or metformin to help prevent DKA or HHS from developing.

Moreover, our analysis of network toxicology provided some insight regarding possible pathways and molecules that may be involved with the toxicities associated with capivasertib: for example, AKT1, IGF1, PTEN, TP53 and GSK3B—were found to be key players in PI3K–AKT pathway. Drug‐target interaction analysis showed significant overlap with key regulators in insulin and IGF1 signaling, providing a biological rationale to explain the significant level of metabolic deregulation that we have reported. There is an apparent physiological upregulation in PI3K/AKT pathway upon IGF1R stimulation. AKT downregulation disrupts the negative loop above and therefore may potentiate insulin/IGF1 signaling at the level of unexpectedly leading to an increase of insulin resistance. Similarly, the negative regulator of PI3K activity (PTEN) that is absent or mutated, could enhance downstream effects of capivasertib and lead to increased sensitivity to the drug in PTEN‐deficient tumors. Moreover, the major downstream targets such as mTORC1, GSK3*β* or FoxO transcription factors are involved in both beneficial (therapeutic) and deleterious (adverse) responses. For example, blocking AKT leads to the de‐phosphorylation of FoxO1 allowing it to translocate to the nucleus where it activates genes involved in hepatic gluconeogenesis, thereby raising the level of sugar in the blood. Meanwhile, AKT suppression in epithelium (e.g., intestines, skin), disrupts cell proliferation/tissue repair processes, increased risk of mucosal injury and cutaneous adverse events.

Several limitations of this study should be acknowledged. First, as a spontaneous reporting system, FAERS is subject to inherent biases (e.g., underreporting, missing data) and establishes only correlative, rather than causal, associations without adjusting for confounders like comorbidities or concurrent medications. Additionally, geographic bias (88% US‐based reports) limits the generalizability of our findings. Furthermore, the high proportion of death outcomes (14.29%) must be interpreted cautiously, as these fatalities cannot be conclusively attributed to capivasertib toxicity rather than to underlying disease progression. Second, our network toxicology predictions rely on in silico analyses, meaning the precise molecular interactions remain hypothetical. Building upon these limitations, future research must prioritize both clinical and experimental validation. Specifically, prospective registry studies are essential to validate the safety signals identified herein, whereas real‐world electronic health record (EHR) data should be leveraged to quantify the absolute incidence of key AEs, such as hyperglycemia. Furthermore, future investigations should explore predictive biomarkers for AKT inhibitor‐induced oral and cutaneous toxicities (e.g., evaluating salivary IGF‐1 levels) and conduct targeted subgroup safety analyses—particularly in patient cohorts harboring specific driver alterations like the *AKT1* E17K mutation—to optimize personalized toxicity management.

## 5. Conclusion

In summary, in this study, we combined AE monitoring with computer toxicity analysis for the discovery of known and novel AEs of capivasertib. Discovery of novel safety issues as well as explanation of their mechanisms at the molecular level offer new insights into the safety profile of adverse reactions of this compound. These findings provide a valuable reference for clinical decision‐making, support proactive risk management, and inform the design of future mechanistic and epidemiological studies to further characterize capivasertib‐related toxicities.

**Nomenclature**
BC: Breast cancer
HR+/HER2−: hormone receptor‐positive, HER2‐negative.
ET: endocrine therapy
FDA: Food and Drug Administration
AE: adverse event
RCT: randomized controlled trial
FAERS: Food and Drug Administration′s Adverse Event Reporting System
PT: Preferred Term
MedDRA: Medical Dictionary for Regulatory Activities
PPI: protein–protein interaction
GO: Gene Ontology
KEGG: Kyoto Encyclopedia of Genes and Genomes
MD: molecular dynamics
GAFF: general Amber force field
RESP: restrained electrostatic potential
NVT: constant number of particles, volume, and temperature
NPT: constant number of particles, pressure, and temperature
PME: particle mesh Ewald
RMSD: root‐mean‐square deviation
RMSF: root‐mean‐square fluctuation
RoG: radius of gyration
SASA: solvent‐accessible surface area
FEL: Gibbs free energy landscapes
scRNA‐seq: single‐cell RNA sequencing
GEO: Gene Expression Omnibus
UMI: unique molecular identifier
UMAP: uniform manifold approximation and projection
GSEA: Gene Set Enrichment Analysis
hdWGCNA: High‐dimensional weighted gene co‐expression network analysis
ROR: reporting odds ratio
PRR: proportional reporting ratio
BCPNN: Bayesian confidence propagation neural network
EBGM: empirical Bayes geometric mean
CI: confidence interval
*χ*2: chi‐squared
IC 025: Information Component 025
SOC: system organ class
DKA: diabetic ketoacidosis
HHS: hyperosmolar hyperglycemic state
EHR: electronic health record

## Author Contributions

Zhanyang Luo and Xingchen Yang conceived the research idea. Zhanyang Luo, Yi Shi, and Bukun Zhu conducted data cleaning and literature review. Zhanyang Luo, Bukun Zhu, Yi Shi, and Qionglian Huang contributed to drafting and critically revising the work for intellectual content. Zhanyang Luo, Bukun Zhu, and Yi Shi, conducted the analysis and created the figures and tables. Wei Zhang, Youyang Shi and Xingchen Yang provided a critical review of the manuscript. All authors have read and approved the manuscript. Zhanyang Luo, Yi Shi and Bukun Zhu have contributed to the work equally and should be regarded as cofirst authors.

## Funding

This study was supported by the National Natural Science Foundation of China, 10.13039/501100001809, 82205114; Research Grant for Health Science and Technology of Pudong Municipal Commission of Health Committee of Shanghai, PW2024A‐81; and Talents Training Program of Fudan University Pudong Medical Center, YQ202408.

## Disclosure

All authors have read and approved the manuscript.

## Ethics Statement

Considering that the FAERS database is publicly accessible, and patient records are anonymous and de‐identified, it does not involve informed consent or ethical approval.

## Conflicts of Interest

The authors declare no conflicts of interest.

## Supporting Information

Additional supporting information can be found online in the Supporting Information section.

## Supporting information


**Supporting Information 1** Table S1. 2 × 2 table for signal detection.

**Supporting Information 2** Table S2. Four major algorithms applied for signal detection.

## Data Availability

All data that supports the findings of this study are available in the supplementary material of this article.
