# Supplementary material for: Pharmacovigilance Signal Detection and Mutually Exclusive Driver Mutations of the PI3K/AKT Pathway in Breast Cancer Treated With Capivasertib
Source: Hum Mutat. 2026 Jun 25;2026:9532614. doi: 10.1155/humu/9532614 (PMC13295149; doi:10.1155/humu/9532614)
Supplement: Supplementary file 1 — Supporting Information 1 Table S1. 2 × 2 table for signal detection. [file HUMU-2026-9532614-s001.docx]

**Table S1. 2×2 table for signal detection.**

| **Type of drug** | **Target adverse** **reaction reports** | **Other adverse reaction reports** | **Sum** |
| --- | --- | --- | --- |
| **Capivasertib** | a | b | a+b |
| **Other drugs** | c | d | c+d |
| **Sum** | a+c | b+d | N=a+b+c+d |

**a**, Number of reports containing both the target drug and target adverse reaction reports; **b**, Number of reports containing other adverse reaction reports of the target drug; **c**, Number of reports containing the target adverse reaction reports of other drugs; **d**, Number of reports containing other drugs and other adverse reaction reports; **N**, The number of reports.
